# From Traditionally Extensive to Sustainably Intensive: A Review on the Path to a Sustainable and Inclusive Beef Farming in Brazil

**DOI:** 10.3390/ani14162340

**Published:** 2024-08-14

**Authors:** Mariana de A. Pereira, Davi J. Bungenstab, Valeria P. B. Euclides, Guilherme C. Malafaia, Paulo H. N. Biscola, Gilberto R. O. Menezes, Urbano G. P. de Abreu, Valdemir A. Laura, Ériklis Nogueira, Rodiney de A. Mauro, Marta P. da Silva, Alessandra C. Nicacio, Roberto G. de Almeida, Rodrigo da C. Gomes, Juliana C. B. Silva, Vanessa F. de Souza

**Affiliations:** 1 Embrapa Beef Cattle, Av. Radio Maia, 830, Vila Popular, Campo Grande 79106-550, MS, Brazil; davi.bungenstab@embrapa.br (D.J.B.); valeria.pacheco@embrapa.br (V.P.B.E.); guilherme.malafaia@embrapa.br (G.C.M.); paulo.biscola@embrapa.br (P.H.N.B.); gilberto.menezes@embrapa.br (G.R.O.M.); valdemir.laura@embrapa.br (V.A.L.); eriklis.nogueira@embrapa.br (É.N.); rodiney.mauro@embrapa.br (R.d.A.M.); marta.pereira@embrapa.br (M.P.d.S.); alessandra.nicacio@embrapa.br (A.C.N.); roberto.giolo@embrapa.br (R.G.d.A.); rodrigo.gomes@embrapa.br (R.d.C.G.); juliana.correa@embrapa.br (J.C.B.S.); vanessa.felipe@embrapa.br (V.F.d.S.); 2 Embrapa Pantanal, Rua 21 de Setembro, 1880, Aeroporto, Corumbá 79320-900, MS, Brazil; urbano.abreu@embrapa.br

**Keywords:** beef farming, Brazil, sustainable intensification, public policies

## Abstract

**Simple Summary:**

Agriculture worldwide has been challenged by how to grow sustainably to feed a rising population without depleting natural resources. This is also the case in Brazil and, in particular, for beef farming, which is carried out across the country in all biomes and plays a relevant role in Brazil’s economy. This article reviews the major changes in beef farming over the last few decades toward a sustainable intensification of grass-fed beef. We also discuss the potential impact of technological developments on small- to medium-scale farms and reflect on some initiatives that may help farmers stay up-to-date with climate-friendly technologies. Their persistence will rely on their ability to remain competitive, which requires the incorporation of sustainable technologies, the development of new capabilities such as digital literacy, and access to credit and technical assistance. This study has implications for policymakers, financial institutions, and extension services.

**Abstract:**

Brazil is the second largest beef producer and a leading exporter, contributing to some 3000 t CWE in global markets (27.7% of market share). The sector has experienced substantial development, but yields remain far below potential, and there are growing concerns regarding land use change and greenhouse gas emissions. The need for sustainable technologies, such as sound pasture management and integrated farming systems, is evident, but adoption may be low amongst farmers unable to keep up with technological advances. This article describes the historical developments of Brazilian beef farming towards sustainability and discusses possible socioenvironmental outcomes. We combined an extensive literature review, public data, and our own insights as senior researchers to achieve that. The trajectory shown here evidenced the technological intensification of Brazilian beef farming, with strong support of public policies for decarbonizing agriculture. Nonetheless, the pace of this transition may affect small to medium farmers with limited access to information, technologies, and credit. Our recommendations involve a broad program of technical assistance and training on sustainable technologies, including financial and digital literacy. A novel approach to financing farmers is suggested to support a sustainable and inclusive transition in beef farming in Brazil.

## 1. Introduction

Brazil stands out in the global beef market, holding the world’s largest commercial herd and leading beef exports, accounting for 27.7% of the market share [1]. The Brazilian beef supply chain has had vigorous growth over the past decade. The sector’s GDP increased from BRL 356 billion in 2011 to BRL 913 billion in 2021 (1.00 BRL = 0.1926 USD = 0.1818 EUR), with an average growth rate of 9.9% [1]. According to Basso et al. [2], in 2023, crops, livestock, and agribusiness as a whole contributed approximately 18%, 6.8%, and 24.8% to Brazil’s GDP, respectively. The number of importing countries has grown to over 145 [1], with China becoming a significant destination alongside other established markets like Hong Kong, the European Union, Egypt, and Chile. The new sanitary status of being free from foot-and-mouth disease without vaccination, achieved by several Brazilian states, has opened new international markets [3]. Domestically, livestock productivity increased by 159% from 1990 to 2020, while pasture area decreased by 13% [4], estimated at 153.8 million hectares in 2022 (Table 1).

Since the 1990s, after market liberalization by the Brazilian government, the beef supply chain has undergone technological modernization, resulting in higher productivity, better meat quality, and greater competitiveness. From 2000 to 2010, slaughter age was reduced to 33 months, heifers’ age at first breeding dropped from 36 to 30 months, weaning rates increased from 57% to 68%, and weaning weight increased from 167 to 190 kg [5]. Favorable climatic conditions, abundant land at relatively low prices, a large labor supply, and the development of tropically adapted technologies significantly contributed to the sector’s boom [4], supported by public policies [6], alleviating previous concerns over beef imports, common until the 1980s. 

However, growth in the sector has not been uniform across regions and types of farmers and remains a significant challenge in reducing inequalities and preventing social displacement. Moreover, modern beef farming must rapidly address environmental issues, such as its link to deforestation and the cattle carbon footprint, both crucial for combating global warming. Considering the crucial role Brazilian beef plays in global food security and its influence on worldwide beef markets [1], understanding the evolution of the country’s sector and the main challenges ahead will better inform policymakers, researchers, and other stakeholders involved in supporting and promoting sustainable farming practices, both domestically and internationally.

This article aims to describe the major changes in Brazilian beef farming in the recent past and reflect on the issues and challenges ahead from a multidisciplinary perspective. For this purpose, we used a mixed approach, combining an extensive literature review based on scientific research and public data to enrich our exploration and insights.

## 2. Historical Development of Beef Farming in Brazil

### 2.1. The Transformation of Cerrado and Expansion to the North

Until the 1960s, cattle herds were predominantly found in the south and southeastern areas of Brazil, under extensive, low-input systems. In the late 1960s, public policies encouraged farmers to “conquer” the Cerrado, a savanna-type vegetation typically found in Central Brazil. Agriculture began moving northward, clearing land for cash crops like dryland rice and soybeans. With the advance of crops, machinery became available for sowing pastures. Palisade grasses from Africa were introduced in the early 1970s, along with Indian cattle (Zebu breeds), creating a favorable environment for cattle farming [6]. The Brazilian Agricultural Research Corporation—EMBRAPA, created in 1973 to lead the National System of Agricultural Research, was strategic for developing tropical technologies adapted to Cerrado’s harsh conditions, which was later considered the “Tropical Agriculture Revolution” of the 20th century [2]. 

**Figure 1:**
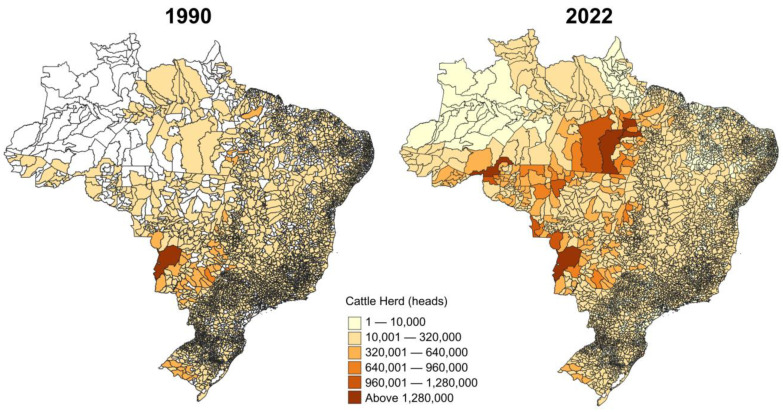
Distribution of cattle herds in Brazil in 1990 and 2022. Source: Prepared by the authors. Adapted from the Brazilian Institute of Geography and Statistics—IBGE.

Cash crops expanded further northwards, followed closely by cattle herds. Soybeans and maize, in particular, but also sugarcane and eucalyptus forestry, became increasingly economically attractive in Central Brazil, especially in fertile soils, constantly pushing cattle farming to fringe areas. Consequently, traditional cattle farming regions, such as southern and southeastern Brazil, gradually saw a reduction in herd size and growth rates [5], while growth has remained robust in northern Brazil. Our own analysis confirms this trend, showing a significant shift of herds from the central to the northern region of Brazil over the last decades (Figure 1).

This shift is not only related to lower land prices in the north and overall improvements in farming techniques adapted to the Amazonian biome [7], but fundamentally to a significant increase in the regional demand for cattle products, like milk and beef, due to population and purchasing power growth [8]. According to official data, the region’s population exceeded 17 million inhabitants in 2022, with Pará, Amazonas, and Rondônia leading the ranking [9]. Pará, Roraima, and Acre are the major suppliers of the regional beef market [5]. More recently, cattle herds have been expanding into the states of Maranhão, Tocantins, Piauí, and Bahia, comprising the so-called “Matopiba”. This new agricultural frontier is evidence of a shift in the axis of production and will continue to do so in the coming decades [7].

Despite ongoing changes, central Brazil remains a significant cattle zone, with Mato Grosso do Sul, Goiás, and Mato Grosso states being particularly relevant [5], as illustrated in the map above (Figure 1, year 2022). According to ABIEC [1], in 2022, the North and Central regions held 20.3% and 35.4% of the cattle herd, respectively. 

### 2.2. The Launch of Improved Tropical Forage and New Husbandry Practices

Grasslands are the primary land use in Brazil (21%), after land spared for conservation and indigenous peoples (40.7%) and for conservation within private farms (25.6%) (Table 2) [10]; the latter two comprise 66.3% of the total territory. Brazilian cattle, from cow-calf to finishing, is essentially grass-fed on extensive sown pasture systems, resulting in one of the lowest beef costs in the world [11]. While not the standard, feedlots and semi-intensive finishing systems can be strategic for improving production and reducing externalities, as they can save a year of extra grazing and help to reduce greenhouse gas (GHG) emissions by accelerating the weight gain and lowering the age at slaughter, therefore, reducing the total emissions per head and per kilo.

Pasturelands play a strategic role in Brazilian beef competitiveness while also allowing for the use of least-prone agricultural land. The introduction of *Brachiaria* grass back in the 1970s, well adapted to the Brazilian climate and soil, was one of the milestones in the development of livestock farming in the country.

Until 1985, natural grasslands were predominant, along with low-input farming systems. About ten years later, they represented only 30% of total Brazilian pastures, mainly due to physical characteristics and cultural traditions (check Box 1 for an example). From the 1980s onwards, the collection of forage genetic resources in Brazil and Africa and the selection process, based on their natural variability or through crossings, set the grounds for the Brazilian tropical forage development program at Embrapa, which led to a substantial increase in animal production in the following decades [12]. Several forage cultivars have been launched and adopted in the most diverse production systems and biomes, including those of the genera *Brachiaria*, *Panicum*, *Andropogon*, *Stylosanthes*, *Arachis*, and *Cajanus*.

Another milestone for beef farming was the introduction of Indian cattle, mainly Nellore, Guzera, and Gyr breeds. Nellore (*Bos indicus*) and its crosses became the main beef breed [13], representing about 80% of the Brazilian herd. Nellore is extremely well-adapted to the country’s conditions [14,15], notably for heat and parasite tolerance, while maintaining the capacity to efficiently use low-nutrition tropical forages. Genetic programs that started in the 1980s improved this performance by progressively shifting from empirical animal selection to fixing racial characteristics to production efficiency [16]. As computational resources evolved, so did genetic evaluation, with the pioneering initiative of Embrapa working with sire models at first and then animal models. Currently, the Embrapa-Geneplus Beef Breeding Program, the Zebu Genetic Improvement Program (from the Brazilian Zebu Breeders Association—ABCZ), and other breeding programs are increasingly incorporating genomic data and innovative selection criteria associated with carcass quality, sexual precocity, feed efficiency, and environmental impact (e.g., water intake, greenhouse gas emissions).

Nellore heifers, however, are late in their development and onset of puberty, with their first parturition occurring, on average, at around 36 months, in contrast with 24 months for European breeds (*Bos taurus*) [13]. The earlier the reproductive life starts, the greater the females’ productivity [17,18], which is a determinant for profitability in cow–calf operations, along with the birth rates. In this context, reproductive bio-techniques have been of particular importance for promoting sound reproduction management, given the prevalence of extensive farming systems in Brazil. Artificial insemination at detected estrus (AIE) and timed artificial insemination (TAI) are the primary tools used to increase production through introducing superior sires in commercial herds, including fertility-related ones. According to ASBIA [19], 23.5% of the 63 million beef cows are currently inseminated in Brazil, mostly with Zebu breeds. By large, TAI is the main method (98% of cases) and results in a 50% pregnancy rate, on average, in each shoot [20]. The annual sales growth rate of synchronization protocols for TAI has been around 32%, in contrast with 6.7% for semen doses.

Other reproductive bio-techniques still face scaling challenges in Brazil. In vitro embryo production (IVP) for embryo transfer (ET), sex-sorted semen, vitrification, and freezing for direct transfer remain niche markets, mostly used in herds with superior genetics. The challenges to increasing the adoption of these biotechnologies include [21]: (i) the lack of a comprehensive understanding of productivity and economic benefits from using such technologies; (ii) an insufficient number of specialists on the field; (iii) the relatively low efficiency of AI and ET programs; (iv) research failing to assess reproduction within a systems approach; and (v) inadequate coordination of the supply chain restraining the communication and transfer of these technologies to farmers, among others. Despite the limitations, these technologies are promising and will eventually reach commercial herds [22]. Brazil is one of the largest producers of in vitro-derived cattle embryos and is the leader in IVP embryo transfers in the world, holding a market share of 37%. The country has a high number of dedicated laboratories and a vast herd of *B. indicus* cows, which are known for higher oocyte recovery, a higher number of viable oocytes, and higher production of viable embryos than *B. taurus* cows [23,24].

Alongside the genetic improvement of cattle breeds, the progress of the nutrition industry has occurred, focusing primarily on the nutrition gap between nutrient poor soils and forage and the increasing nutrient demand by genetically improved cattle. In the 1970s, mineral deficiencies in Brazilian soils were identified as one of the major reasons for mineral undernutrition-related diseases, commonly found in cattle [25,26]. In the following decades, a consistent nutrition industry emerged across the country, promoting adequate mineral nutrition by developing and commercializing mineral mixtures that helped tackle cattle deficiencies mainly of phosphorus, sodium, copper, and zinc. It allowed for significant improvements in growth rate, carcass quality, fertility, and health. Furthermore, a number of new nutritional strategies based on protein, energy, and feed additive supplementation [27] were developed and adopted, minimizing the performance loss in the dry season and, occasionally, improving it in the rainy season. 

With the expansion of Brazilian agriculture and the structuring of the grain processing industry, larger amounts of cereals and co-products became available in the market [28], which favored the growth of feedlot operations that finish about 7.6 million cattle [1] a year. Another 5 million heads are estimated to be finished with high-grain diets (e.g., 1.5% to 2% intake of concentrates relative to their body weight), but in combination with grazing, namely “semi-intensive finishing”. Considering that 42.3 million heads were slaughtered in Brazil in 2022 [1], intensive and semi-intensive finishing systems represented around 30% of the total; the remainder was cows (over 42% of slaughter in 2023), culled animals, and dairy cattle. Since these intensive systems are mainly for male cattle and are usually used for a short period (100 days on average) to make them grow from 400/450 kg to 570 kg of body weight, the carcass gain amounts to up to 30% of the final carcass weight. As intensive and semi-intensive finishing systems are becoming more widely adopted, the carcass weight is increasing (i.e., from 228.5 kg in 2011 to 257.3 kg in 2022) [1] due to the higher energy content in finishing diets [5].

Such outstanding development of the Brazilian beef sector was only possible with the support of public policies, farmers’ private investments, and better coordination of the supply chain. Between the 1950s and 1990s, several public policies encouraged the occupation of Central Brazil through agriculture [29]. The farms’ profitability relied more on asset valuation due to inflation and herd/land expansion than on productivity [30]. More recently, policies shifted to shape the future of Brazilian farming towards a sustainable intensification of production systems. We present the two most influential policies below. 

### 2.3. Key Public Policies for Recent Developments in Brazilian Agriculture

#### 2.3.1. The Forest Code

The Brazilian Forest Code was established in 1934, revised in 1965, and reformulated in 2012. The so-called “Brazil New Forest Code” sets boundaries for the use and protection of private land in Brazil [31]. The Code mandates that farms set aside areas for preservation and protection as Permanent Protected Areas (PPAs) or Legal Reserves (LR). PPAs are critical environmental areas that must remain intact, whereas LRs are areas covered by native vegetation that can be sustainably managed but not cleared. The size of PPAs varies based on geographical features and physical attributes (e.g., size of water bodies; slope steepness), while the size of LRs ranges from 20% to 80%, depending on the biome and type of vegetation. In the Legal Amazon, farms must maintain 80% as LRs if in typical forest areas, 35% if in Cerrado areas (savannah type), and 20% if in grassland areas; outside the Legal Amazon, 20% must be maintained as LRs, irrespective of the type of vegetation (for more information on the Legal Amazon, see [31]). PPAs and LRs are not exchangeable, and where both exist, PPA areas must be added to the LR. As shown in Table 2, over 280 million ha are preserved within Brazilian farms, representing 33.2% of the territory [10]. Together with other conservation and preservation areas, the total preserved area amounts to 66% of the country.

Another critical compliance requirement of the New Forest Code is the enrollment of landowners in the Rural Environmental Registry (CAR, in Portuguese). This registry involves a self-declaration of georeferenced data on land use within farms, allowing governments to monitor deforestation and better plan rural areas while enabling farmers to access public services and policies, such as environmental grants and rural credit. However, the full implementation of the CAR has been challenging, with progress slow in some states.

#### 2.3.2. The ABC Program and the ABC+ Plan

In 2010, the Brazilian government launched the Low Carbon Agriculture Plan (ABC Plan), a greenhouse gas (GHG) emission mitigation strategy for the agricultural sector. The plan, expressed through Nationally Appropriate Mitigation Actions (NAMAs) to the Secretariat of the United Nations Framework Convention on Climate Change (UNFCCC), aimed to voluntarily reduce GHG emissions by 37% by 2025 and 50% by 2030, relative to 2005 levels [32]. Following the 2015 Paris Agreement, Brazil ratified and expanded its decarbonization targets with its Nationally Determined Contributions (NDCs).

Investments in the first phase of the plan (2010–2020) exceeded USD 3.5 billion, promoting low-carbon technologies such as recovery of degraded pastures, biological nitrogen fixation, no-tillage, integrated crop–livestock–forestry systems (ICLFS), agroforestry, planted forest, and livestock waste management. The proportion of pastures with some level of degradation decreased from 71% in 2010 to 58% in 2018 [33]; beef productivity in Brazil increased from 52 to 63 kg of carcass ha^−1^ between 2012 and 2020 [1]; areas with any combination of ICLFS expanded from 2 million ha in 2009 to 17 million ha by 2020/2021 [34], exceeding the original target of 4 million ha [32]. Given these results, the ABC plan was extended until 2030 with the launch of the new “Sectoral Adaptation Plan for Low Carbon Agriculture for Sustainable Development” (ABC+ Plan). This new cycle includes irrigated systems, intensive cattle finishing, and the use of bio-inputs [35]. Given the potential of ICLS for diversifying and intensifying beef production while promoting GHG mitigation and improved water use [36,37], adoption of these production systems is expected to reach 35 million ha by 2030 [34].

Additionally, in December 2023, the Ministry of Agriculture and Livestock announced the National Program for the Conversion of Degraded Pastures into Sustainable Agricultural, Livestock, and Forestry Production Systems (Decree 11.815), which promotes good agricultural practices to increase carbon capture [38]. The program’s objectives include converting up to 40 million ha of degraded pastures into crops, planted forests, or improved pastures, reducing deforestation pressure, and contributing to food, feed, and energy security while encouraging financial institutions and the capital market to develop financial opportunities.

### 2.4. Sustainable Intensification: The Path to Brazilian Low-Carbon Beef

**Figure 2:**
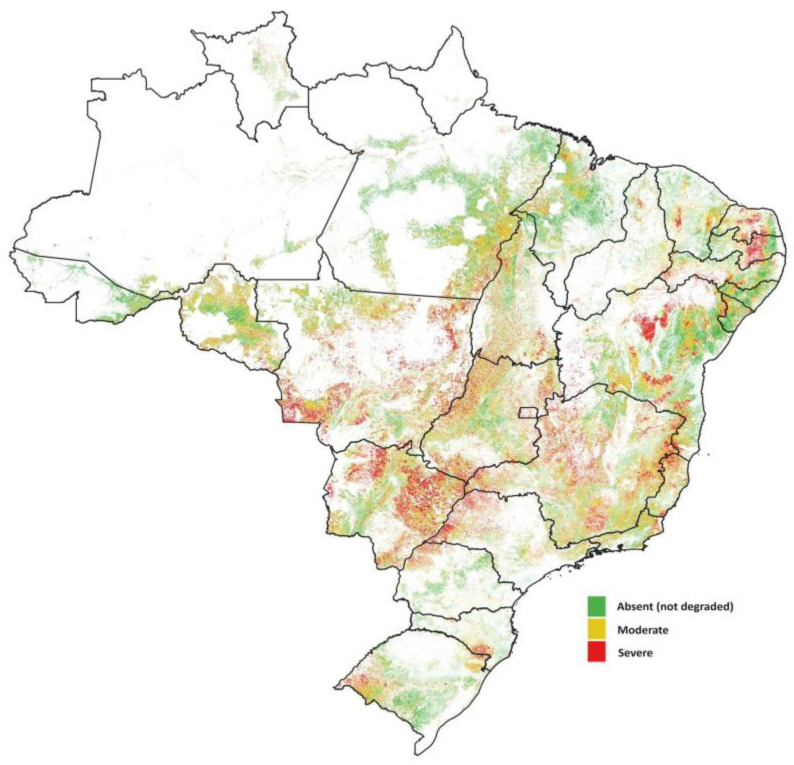
Pastures in Brazil according to three levels of degradation, 2022. Source: Prepared by the authors, based on the Lapig database [39].

Brazil has approximately 154 million ha of pasture, of which 110 million ha present some level of degradation (about 60% of the total) [39] (Figure 2). Pasture degradation primarily results from increasing grazing pressure and poor pasture management, posing significant economic and environmental challenges [40,41,42]. In response, there is growing appeal for the sustainable intensification of Brazilian agriculture, and beef farming in particular. Beef production intensification can reduce greenhouse gas (GHG) emissions per kilo of product (i.e., emission intensity) through a supply of improved and balanced diets. These diets include a higher proportion of grains and supplements with the potential to reduce methane emissions, increase daily weight gains, reduce cattle age at slaughter, and increase pasture carrying capacity by removing temporarily grazing animals from some paddocks [43]. Although there may be some concerns about the environmental consequences of producing more cereals for cattle finishing, further expansion of grain production should come from increased productivity (i.e., continuing the upward trend) [2] and from using other consolidated areas, such as low-productivity pastures (see [42,43] for more details) or livestock–crop integration [36,37].

For ease of analysis, pasture degradation has been defined by three levels—absent (not degraded), moderate, and severe [39] (Figure 2)—relative to ideal yields for each pasture species [41]. According to Bolfe and collaborators, there are 28 million ha of moderately or severely degraded pastures, covering suitable land for crop production [40]. 

The higher the degradation level, the greater the recovery cost [42], which can be prohibitive for some farmers. Recovering 30 million ha of pasture for improved beef farming would require investments of USD 8.6 billion and could yield returns ranging from USD 440 million to USD 6.7 billion, depending on various beef price scenarios [42]. It is noteworthy that the development of beef farming has spillover effects on regional prosperity, as evidenced by the Human Development Index (HDI) in a study by Lima et al. (2023) [15]. 

Alternatively, converting 28 million ha of degraded pasture into crop production, as proposed by the National Program for the Conversion of Degraded Pastures [38], could potentially increase by 35% the total crop area in relation to the total planted area in 2022/2023 crop season [40]. Given the high costs associated with recovering degraded pastures [42], farmers may opt for an integrated crop–livestock system (ICLS) [40,41,42,43,44]. Integrated farming systems, such as ICLS and ICLFS, when including forestry (F), can enhance yields, food security [44], profitability, and reduce economic risks through production diversification [45] while also creating new income opportunities, such as payments for environmental services and carbon credits [46].

A comprehensive literature review carried out by Rodrigues and collaborators showed that ILFS are low-carbon agriculture models that provide ecosystem services related to nutrient cycling, biodiversity, and soil erosion control [47]. These benefits arise from the synergistic effects of ICLFS components on space and/or time. Integrating trees into the system allows for an average carbon accumulation of 30 kg tree^−1^ year^−1^ (equivalent to sequestrating 110 kg of CO_2_ tree^−1^ year^−1^) and enhances animal welfare by providing shade and shelter to cattle. These production systems are also more resilient under global warming conditions compared to specialized farms [44]. It is important to note that these models can be adapted to any biome, climate condition, farmer type, and market, although limitations such as infrastructure deficits and low farmer access to technical assistance and credit can hinder adoption [48].

To encourage the adoption of sound agricultural practices, including ICLFS, and to add value to beef, Embrapa has been developing beef farming protocols since 2005. Recently, concerns over beef GHG emissions have prompted the protocols to focus more specifically on this issue. As a result, Embrapa launched Carbon Neutral Brazilian Beef—CNBB [49] and the directives for Low Carbon Brazilian Beef—LCBB [43], both aimed at increasing productivity while neutralizing or reducing methane emissions, respectively, by improving pasture management and applying Good Agricultural Practices (GAP), with or without planted trees. However, due to institutional challenges, the uptake of CNBB remains low, and the LCBB protocol is going to be launched in 2024. 

Given Brazil’s renewed commitments to reduce GHG emissions by 50% by 2030 and achieve carbon neutrality by 2050 [50], addressing the challenges of implementing the aforementioned policies, programs, and protocols is crucial. Overcoming barriers, including sociopsychological factors such as cultural resistance, infrastructural issues like the absence of paved roads, bridges, and warehouses, and institutional obstacles such as limited access to technology, credit, and technical assistance, is essential for further adoption of low-carbon technologies.

## 3. Discussion: Social Inclusion and Other Challenges for Brazilian Beef Farming

**Figure 3:**
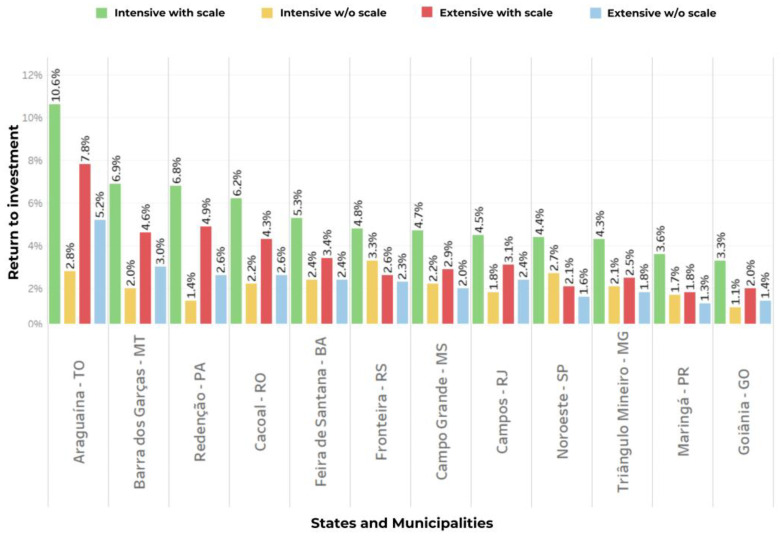
Return on capital (%) of rearing and fattening cattle with and without scale (below 500 animal units) and at different levels of intensification in selected municipalities in Brazil, 2019. Source: Adapted from [53].

Despite the significant growth of the Brazilian cattle sector and high expectations for further developments, numerous challenges persist. Arango and collaborators draw attention to the governments’ struggles related to “reconciling the goals of benefiting from business and livelihood opportunities associated with cattle production while reducing [their] GHG emissions” [48] (p. 2). Given the size and dispersion of the Brazilian cattle herd, in addition to various farmers’ typologies, this challenge is further amplified by uneven access to technology and knowledge. Over half of Brazil’s five million rural establishments have cattle [51], dispersed throughout the country [15]. Approximately 76% of these are smallholder farmers, who account for 31% of the total herd. These farmers often have little or no access to rural credit and technical assistance [30], making them particularly vulnerable to rural poverty [52]. The small scale of production also impacts the return on capital investment [53], perpetuating a cycle that keeps many of these farmers in a poverty loop, as depicted below (Figure 3). 

Rising costs, driven by increased labor expenses and land appreciation, coupled with growing socioenvironmental restrictions [54,55] are adding pressure on the farms economic margins. Reports from Athenagro Rural Consultancy [56] indicate that beef farmers with varying levels of technology started 2024 with cash reserves ranging from BRL 300/BRL 400 ha^−1^ (USD 57.8/USD 77.0) to BRL 750/BRL 1200 ha^−1^ (USD 144.5/USD 231.1), depending on whether the farms had low or high technology uptake, respectively. The reports also revealed that high-technology farms accumulated deflated profits of BRL 25,000 to BRL 40,000 ha^−1^ (USD 4815–USD 7704) over the last 25 years, while low-technology farms only managed between BRL 5000 and BRL 10,000 ha^−1^ (USD 963–USD 1926) during the same period. These figures serve as proxies for the farmers’ investment capacity to keep pace with sectoral technological advancements, suggesting that low-technology farms are at risk of market exclusion.

Many low-technology farms are smallholdings, and their persistence is frequently associated with public policies specifically targeting this group [53], such as: (i) PRONAF—a low-cost credit for eligible family farms, that is, farms with less than four fiscal modules (i.e., variable area, according to the region), primarily reliant on family labor and management, in which most income comes from farming; (ii) minimum price policies; (iii) rural retirement pensions; (iv) allowances, like *Bolsa Familia* (“Family Allowance”—free translation), which is a public policy targeting rural and urban families whose members earn less than USD 44 per month each; and several others [57]. Specifically about PRONAF, the main tool to finance smallholder farmers towards a sustainable transition, Abath [58] points out that the policy fails to provide credit associated with training, technical assistance, and monitoring, which favors the misallocation of resources. It also requires several formal documents, including land title and proof of collateral, that are not always available or too difficult for small farmers to provide, preventing them from accessing the credit lines. These factors also affect small farmers in other countries [48,49,50,51,52,53,54,55,56,57,58,59]. To overcome these limitations, an alternative credit system (i.e., microfinance) could be implemented, whereby smallholding farmers with low or no collateral can borrow increasing amounts of capital at low cost as they pay off prior borrowings while progressively building their financial reputation with the banks or other financing institutions (e.g., cooperatives, farmers’ associations). Microfinance institutions enable farmers to increase their production capital while running low risks [59]. 

In addition to economic factors, other aspects are important in explaining farmers’ decisions on technology uptake [48,49,50,51,52,53,54,55,56,57,58,59,60], such as the characteristics of technology itself and a range of personal, social, and cultural factors [61], making it strongly context-sensitive. Westbrooke and Nuthall argue that small farmers’ personalities, personal characteristics, and objectives play a significant role in their decisions to maintain or expand their farms (e.g., increase area or intensify the production system) [62]. In Brazil, the farms’ objectives vary widely, spanning from subsistence to corporate farms, from low-input and traditional farming to precision farming that is highly market-oriented. Considering the diversity of contexts in which cattle are raised (e.g., six biomes), it is not surprising that production systems and results are so different. These aspects are often overlooked in research, particularly in Brazilian beef farming [60]. 

Despite the differences, Brazilian production models, in general, have been shifting toward more capital-intensive technologies (i.e., “land-saving” technologies) that offer better technical and economic performance [30,31,32,33,34,35,36,37,38,39,40,41,42,43,44,45,46,47,48,49,50,51,52,53,54,55,56,57,58,59,60,61,62,63] and have lower environmental impact than traditional extensive systems [64]. Key technologies for the sustainable intensification of beef production include ICLS and ICLFS, new forages, further genetic improvement of the herds, sound pasture management or recovery, feed supplementation, and good agricultural practices in general [65]. ICLS and ICLFS, in particular, have great potential to transform Brazilian agriculture, as they promote training, higher agricultural income, and better employment opportunities [66], in addition to increasing and diversifying agricultural production with lower environmental impacts. However, barriers to the adoption of such systems among beef farmers are anticipated, including cultural aspects [48,49,50,51,52,53,54,55,56,57,58,59,60,61,62,63,64,65,66,67]. Suggestions to overcome some of these barriers may include highlighting the profitability of the systems [67]; partnerships between beef and crop farmers [68,69]; and shared risks and increased economic viability of integrated systems through farmers associations or cooperatives [70].

With the ongoing impacts of climate change, additional stress has been placed on production systems and their ability to generate regular income. The prospect of increasing beef production with less pasture, as prompted by the Federal Government [38] and less labor [4], requires a transition from extensive to sustainably intensive beef farming reliant on technology adoption and on a supportive innovation system capable of accommodating the various farmer typologies that exist in Brazil. If decoupled from deforestation, Brazilian beef production has the potential to lower greenhouse gas emissions [71], given the recovery of moderately to severely degraded pasture, enhancing carbon sinks [72]. 

In order to encourage technology uptake, it is important to develop an extensive program of technical assistance and training to provide farmers and workers with new capabilities and enhance social capital in sustainable farming [58]. Financial and digital literacy, for instance, will become increasingly important for further improvements in the sector. Without technical and economic knowledge, limited resources can be wasted and few results can be achieved. Considering financial resources are scarce in Latin America [48], efforts should be directed and carried out by both the private and public sectors to be effective and to reach even those farmers in remote areas of Brazil. Many farmers, particularly small and medium landholders, may be unable to implement or maintain sustainable technologies if they continue to struggle to access external funding [56]. Whether Brazil will be able to transition toward a sustainable intensification of beef farming in an inclusive manner remains an open question.

Box 1 **Cattle ranching in Pantanal—a special case of sustainable beef production.**

**Figure 4:**
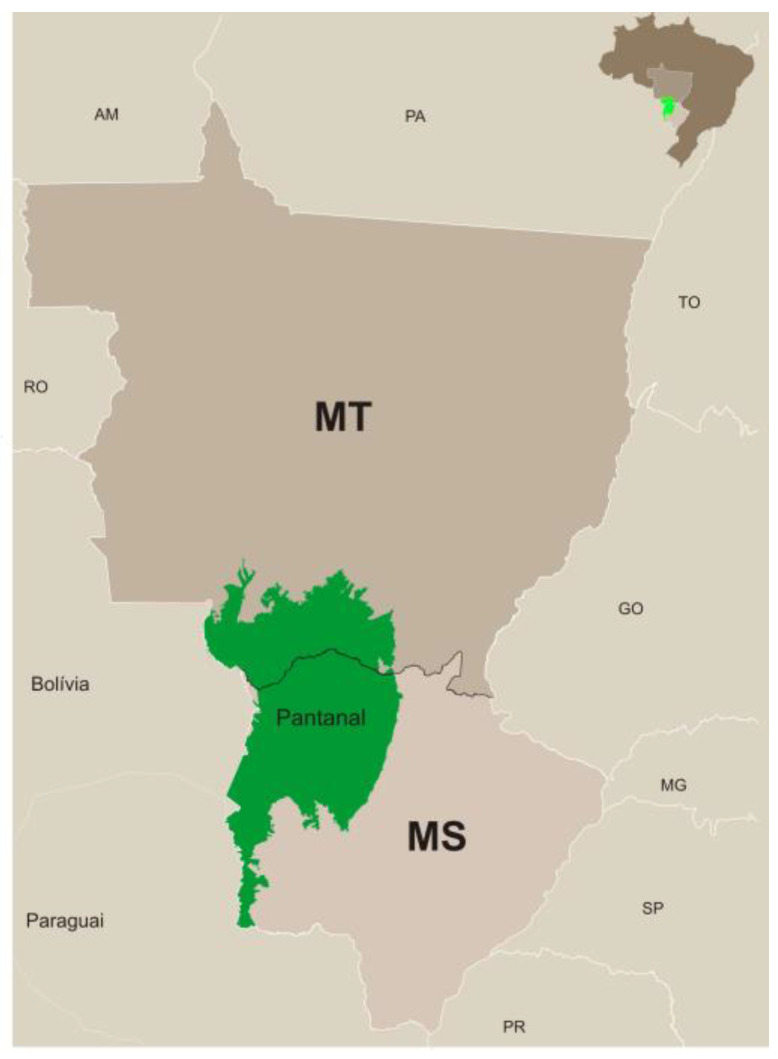
Map of the Pantanal biome (in green), Brazil.

The Brazilian Pantanal biome is a 138,183 km^2^ floodable area, with 65% in the state of Mato Grosso do Sul and 35% in the state of Mato Grosso (Figure 4). It expands into Bolivia and Paraguay, where it is known as the Chaco. It is the largest freshwater wetland in the world, a seasonally flooded lowland fed by the tributaries of the Paraguay River. Pantanal presents a great diversity of landscapes, associated with types of soil, degree of flooding, and the influence of the adjacent biome. Farming in this biome is challenging and unique.

Natural grasslands are the basis of beef cattle ranching in Pantanal. Among the domestic animals introduced by Spanish and Portuguese people are cattle (*Bos taurus*), donkeys (*Equus asinus*), and horses (*E. caballus*). Cattle farming in the Pantanal expanded significantly after the end of the gold panning era in the 19th century, spreading across the natural grasslands of the plain and remaining the main economic activity in the region.

Originally, the herd consisted primarily of *Bos taurus taurus*, but it is now predominantly comprised of Zebu breeds, especially Nellore (*Bos taurus indicus*), due to the beef industry’s preference—a process facilitated by the construction of the Northwest railway line in Brazil [73]. The breed’s rusticity and adaptation to extreme weather conditions such as heat, periodic floods, and severe droughts have favored its adoption. Crossbreeding with Angus, Brangus, and Braford breeds can also be found on the edges of the plain. The area occupied by livestock has grown almost fourfold from 1985 to 2023. Despite this growth, Pantanal retains a coverage of natural vegetation of between 80 and 85% [74,75].

Approximately 93% of Pantanal’s area is privately owned, and, unlike other biomes, it is predominantly characterized by large-acreage farms. About 12% of the farms encompass an area equal to or greater than 10,000 hectares (56% of the total Pantanal area), and 69% have areas ranging from 1000 to 10,000 hectares (43% of the total area) [76]. The stocking rate ranges from 2.5 to 4.2 hectares per head [77], reflecting the predominant extensive grazing system on natural grasslands [78]. Pantanal serves as a net provider of calves to highland areas, focusing on cow-calf farming [71,78], with cattle management adapted to the flood regime [79].

Farmers have gradually been improving native pastures by introducing African grasses, which increase the carrying capacity of Pantanal farms, thus enhancing typically low yield rates. Other technologies for farm management, cattle reproduction, nutrition, and genetics have enabled further efficiency improvements [80]. Interestingly, the objective of Pantanal conservation depends on strengthening the sustainable development of traditional cattle farming in the region [80]. Due to its importance in the regional economy and the local tradition of natural resource conservation, Pantanal cattle farming can provide high levels of sustainability [81]. This is further enabled by the low population density in Pantanal (around 30,000 people), which prevents greater pressure on natural resources.

There is an opportunity to increase the efficiency of cattle production in the biome by maximizing sustainable profit per hectare. However, this must come from the adoption of locally designed technologies for sustainable intensification [82] and should consider multiple socioeconomic aspects as well as different environmental dimensions [81]. Various projects have been carried out in “real farming” systems to enable the sustainable intensification of cattle farming in the region [20,80,83,84]. However, traditional methods of technology transfer have not been effective in increasing adoption. Therefore, there is a need for novel strategies to engage farmers locally.

## 4. Conclusions

In this article, we have highlighted the recent developments of the Brazilian beef sector and reflected upon the socioenvironmental challenges ahead as a result of a transition from extensive to more intensive farming systems through an increased adoption of climate-friendly technologies. Therefore, our work contributes to the body of literature exploring the impacts of sustainable agriculture on society and ecosystems.

Beef farming is an important activity in rural Brazil, both from an economic and a social perspective, as it employs many people in the production and throughout its agrifood chain. It also provides nutritious food and by-products for the Brazilian and international markets, contributing to improving food security and the trade balance. However, the environmental impacts and the technological inclusion of small to medium farms are concerns that need to be addressed.

Further progress is expected, but it may preclude some subgroups of farmers from staying up-to-date with modern technologies, including digital technologies and precision livestock. For instance, how will the new traceability system, currently under discussion, affect small to medium farmers? Would they be able to cope on their own, or would additional strategies and supporting conditions be needed? Further research should address these issues specifically.

For policymakers, it is crucial to recognize and consider some negative externalities of the technological intensification of beef production and of the stricter environmental regulations in order to design sound strategies to include disadvantaged farmers. This may comprise alternative credit systems, human capital building, and a supportive innovation system. Better infrastructure is also needed to allow for better commercialization channels, both for inputs and outputs, access to markets and services, and communication systems. Ultimately, the speed of the sustainable intensification of the beef sector will be closely associated with the level of investments made in agriculture in the years to come.

## Figures and Tables

**Table 1:** Brazil’s beef sector in 2022.

Categories	2022
Area (million hectares)	153.8
Herd (million head)	202.8
Stocking rate (head/ha)	1.32
Meat Production (million t CWE)	10.8
Import (million t CWE)	0.8
Export (million t CWE)	3.0
Internal Availability (million t CWE)	7.8
Population (millions of inhabitants)	214.1
Availability Per Capita (kg/person/year)	36.7

Source: ABIEC Beef Report 2023 [1].

**Table 2:** Areas allocated to the preservation of native vegetation and other land uses in Brazil (2021).

Category of Land Use	Area (ha)	% of Brazilian Area (2021)
Native Vegetation Preserved in Rural Areas	282,858,849	33.2
Full Conservation Units	80,086,542	9.4
Indigenous Peoples Reserves	117,573,859	13.8
Native Vegetation on Unclaimed or Unregistered Areas	84,346,464	9.9
Native Pastures	68,158,758	8.0
Sown Pastures	112,461,952	13.2
Crops	66,454,790	7.8
Commercial Forestry	10,223,813	1.2
Infrastructure, Cities, and Others	29,819,457	3.5
Total	851,984,484	100

Source: Adapted from [10].

## Data Availability

The datasets we used to build our maps are publicly available at the following links: https://www.ibge.gov.br/explica/producao-agropecuaria/bovinos/br (Figure 1—Cattle herd) Accessed on 24 July 2024; https://lapig.iesa.ufg.br/p/38972-atlas-das-pastagens (Figure 2—Pasture distribution with different levels of degradation) Accessed on 20 July 2024. We used ChatGPT v. 4.0 for reviewing and final editing of this manuscript, using the following prompt: Edit for clarity, conciseness, and grammatical accuracy, keeping the structure and sequence of ideas intact as per your instructions.
